# A Fast Analysis Method for Blue-Green Laser Transmission through the Sea Surface

**DOI:** 10.3390/s20061758

**Published:** 2020-03-22

**Authors:** Liwei Dong, Ni Li, Xinhao Xie, Chenying Bao, Xiaolu Li, Duan Li

**Affiliations:** 1 School of Automation Science and Electrical Engineering, Beihang University, Beijing 100191, China; vivian_keith@buaa.edu.cn (L.D.); lini@buaa.edu.cn (N.L.);; 2 State Key Laboratory of Virtual Reality Technology and Systems, Beihang University, Beijing 100191, China; 3 School of Instrumentation and Optoelectronic Engineering, Beihang University, Beijing 100191, China; 4 Beijing Advanced Innovation Center for Big Data-Based Precision Medicine, Beijing 100191, China

**Keywords:** blue-green laser, transmission characteristics, airborne LiDAR, wave model, sea surface, refraction angles, transmittance

## Abstract

The fast estimation of blue-green laser transmission characteristics through the fluctuating sea surface, such as refraction angles and transmittance, is very important to correct operating parameters, detection depth and anti-detection warning in airborne Light Detection and Ranging (LiDAR) applications. However, the geometry of the sea surface is changed by complex environment factors, such as wind and wave, which significantly affect the rapid acquisition of the blue-green laser transmission characteristics. To address this problem, a fast analysis method is provided to rapidly compute the blue-green laser transmittance and refraction angles through the fluctuating sea surface driven by different wind directions and speeds. In the method, a three-dimensional wave model driven by the wind was built to describe the wave spatial distribution varying with time. Using the wave model, the propagation path of the scanning laser footprint was analyzed using the proposed meshing method, thus the transmittance and refraction angles of the optical path can be fast obtained by using parallel computing. The simulation results imply that the proposed method can reduce the time consumption by 70% compared with the traditional analytical method with sequential computing. This paper provides some statistical laws of refraction angles and transmittance through the fluctuating sea surface under different wind conditions, which may serve as a basic for fast computation of airborne LiDAR transmission characteristics in complex environments.

## 1. Introduction

Airborne Light Detection And Ranging (LiDAR) is an effective active detection technique for underwater targets [1]. The airborne LiDAR emits high-power blue-green laser pulses to the underwater target then collects the laser pulses scattered by the target. The position, geometric structures, and physical properties of the target can be calculated by processing the collected pulses [2]. The transmission characteristics of the blue-green laser through the sea surface are the most important factors of detection range and accuracy, which attenuate the energy and change the direction of the blue-green laser pulse. The geometry of the sea surface is changed by the effects of complex environmental factors, such as the fluctuations caused by the dynamic wind. As a result, part of the laser energy will be lost and the propagation path of the laser will deviate during the transmission through the sea surface, significantly affecting the detection range and accuracy. Therefore, it is essential to analyze blue-green laser transmission through the rough sea surface under the influence of complex environmental factors. 

Generally, an airborne LiDAR quickly scans the surveillance area during flight. Meanwhile, the target constantly moves underwater, which makes the transmission through the sea surface more complicated. Thus, dynamic changes of the sea surface and the scanning process of the LiDAR need to be considered to accurately analyze the transmission. The transmission characteristic in the current complex environment is expected to be quickly estimated to undertake corresponding correction and anti-detection. Therefore, it is very important to fast analyze the blue-green laser transmission through the rough sea surface in the complex environment in airborne LiDAR application.

Previous research has focused on the transmission characteristics in the air-sea hybrid transmission path. The turbulent effects on laser beam propagation in the air-sea two-stage links are analyzed with numerical simulation methods [3]; However, the study did not take into account media attenuation or water surface distortions. The spatial multipath effect of the uplink of Airborne Laser Submarine Communication (ALSC) is calculated based on the semi-analytic Monte Carlo method [4]. However, the simulation in this study assumed that the optical performance of each subprocess was stable during the laser propagation process, and it ignored the seawater wave and hidden surge on the sea surface. The optical transmission characteristics on the rough wind-driven sea surface have been studied so far. The influence of the ocean’s optical properties and wind-induced sea surface foam on the shortwave planetary albedo of the ocean-atmosphere system was studied using the Monte Carlo method [5]. Moreover, an analytical solution for radiative transfer was presented in the coupled atmosphere–ocean system with a rough air–water interface [6]. However, these studies focused on the effects of the surface roughness on solar radiation rather than the directional high energy laser pulse in a specific frequency band, and the transmission problem was rarely involved. Some previous research investigated the contribution of the sea surface interface on the laser pulse propagation in various aspects. The spatial and temporal downwelling irradiance profiles below the wavy sea surface was determined with an experimental method [7]. Monte Carlo ray tracing simulations were used to measure refraction angle variation as a result of laser beam interaction with the surface waves [8]. However, only low wind speeds were considered in these studies, and the transmission in high wind speed was not analyzed. The propagation characteristics of the blue-green laser through the sea surface was discussed based on the empirical formulas in a related study [9]. However, the study did not consider the actual sea surface fluctuation and the specific laser detection scene. The effect of wave patterns on refraction and subsequently on coordinate accuracy in airborne LiDAR bathymetry(ALB) was investigated based on the simulations of typical wave patterns [10]. However, the polarization azimuth of the LiDAR system was ignored and the computation time consumption was not controlled in the simulation method.

In conclusion, for airborne LiDAR application, it is necessary to quickly investigate the blue-green laser transmission through the fluctuating sea surface under the influence of complex environmental factors. In this paper, for airborne LiDAR application, a fast method to analyze the blue-green laser transmission through the wind-driven rough sea surface is proposed, which can quickly obtain the transmission characteristic under different wind speeds and directions. In the method, firstly, three-dimensional wave models driven by the sea wind were simulated based on the wave spectrum and the linear wave theory. Secondly, aiming at the circular scanning mode used in airborne LiDAR, the footprints on the dynamic rough sea surface were analyzed and modeled; Finally, the refraction angles and transmittance of a series pulse spots were quickly computed using the meshing method and parallel computing method. 

The remainder of the paper is organized as follows. In Section 2, the modeling and fast method to compute the refraction angle and transmittance through the rough wind-driven sea surface in the actual detection scene are introduced. The simulations and results mainly concerned with the quantitative statistics are presented in Section 3. In Section 4, the simulation results are discussed in the perspective of the specific simulation conditions and actual situations. Section 5 is the summary and conclusion of this work.

## 2. Materials and Methods

### 2.1. Modeling Three-Dimensional Dynamic Wave

Due to the dynamic influence of sea wind, the wave inclination of the undulating and rough sea surface changes continuously, which directly affects the transmission process of laser beam on the sea surface, and causes the complicated variation of transmission energy on the interface. In order to simulate the transmission process of the laser beam on the rough interface, the height and normal of the wave surface must be obtained. In this section, the wave spectrum theory and linear superposition method were used to build a three-dimensional dynamic wave model. Next, we divided the simulated wave surface into meshes, then obtained the heights and normal of every mesh nodes by calculating the wave model.

#### 2.1.1. Wave Model

In oceanography, wave spectrum is the main way to describe the wave motion, a complex random process. The wave spectrum is obtained by using the spectrum analysis method to analyze long-term observed wave elements such as wave height and period. It describes the frequency distribution of wave energy as a random process relative to its constituent waves.

Multiple kinds of wave spectrum have been proposed. The one we use to simulate wind-driven dynamic wave is the Pierson-Moscowitz (PM) spectrum, which is widely used because of its better data base and higher accuracy. The spectrum function is denoted as $S_{PM}\left(\omega \right)$ and described by [11]:

(1) $$S_{PM}\left(\omega \right)=\frac{8.1\times 10^{-3}g^{2}}{\omega^{5}}\exp\left[-0.74{\left(\frac{g}{u\omega }\right)}^{4}\right]$$

where ω is the angular frequency of the wave, g is the gravitational acceleration, u is the wind speed at 19.8 meters above the sea, and the spectral peak frequency is denoted as $\omega_{m}$ and described as

(2) $$\omega_{m}=8.565/u$$

The actual wave surface is three-dimensional, which means that its energy is not only distributed in a certain frequency range, but also in a certain direction range. However, the PM spectrum is a kind of one-dimensional spectrum that can only describe the distribution of energy with frequency. Therefore, a directional spreading function D(θ) was defined to more accurately describe the energy distribution of three-dimensional waves, which is denoted as S(ω,θ) and shown as follows.

(3) $$S\left(\omega ,\theta \right)=S_{PM}\left(\omega \right)\cdot D\left(\theta \right)$$

In this paper, the directional spreading function proposed by the International Towing Tank Conference (ITTC) [12] is used and shown as:

(4) $$D\left(\theta \right)=\frac{2}{\pi }\cos^{2}\left(\theta \right),\left|\theta \right|\leq \pi$$

where θ denotes the angle between the wave and the wind direction above the sea, which is defined as the direction angle of the wave.

Substituting Equations (1) and (4) into Equation (3), the complete energy distribution spectrum can be obtained as:

(5) $$S\left(\omega ,\theta \right)=\frac{8.1\times 10^{-3}g^{2}}{\omega^{5}}\exp\left[-0.74{\left(\frac{g}{u\omega }\right)}^{4}\right]\cdot \frac{2}{\pi }\cos^{2}\left(\theta \right),\left|\theta \right|\leq \pi$$

**Figure 1:**
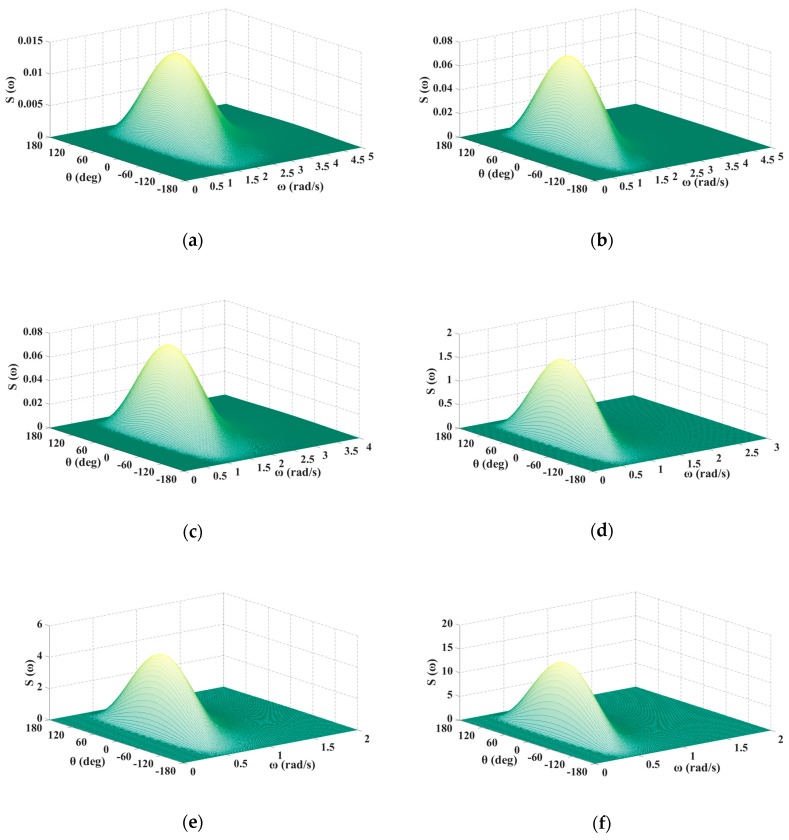
Energy distribution of waves at different wind speeds. (**a**) The wind speed is 5 m/s; (**b**) the wind speed is 7 m/s; (**c**) the wind speed is 9 m/s; (**d**) the wind speed is 13 m/s; (**e**) the wind speed is 16 m/s; (**f**) the wind speed is 20 m/s.

According to Equation (5), energy distribution of waves at different wind speeds were drawn and are shown in Figure 1. It can be seen that most of the energy of waves is concentrated in a certain frequency range, and the range moves to the low frequency part with the increase of the wind speed. Meanwhile, in terms of direction angle, most of the energy of waves is approximately concentrated in the angle range [−90°,90°]. According to the linear wave theory, the wave can be regarded as a stationary random process composed of multiple cosine waves with different periods, frequencies, and directions. Therefore, we only needed to apply the linear superposition method in the range of wave energy concentration, which can effectively represent all waves to quickly simulate the three-dimensional wave.

The geometry of the sea surface is described in a right-hand three-dimensional coordinate frame. The x−y plane denotes the horizontal datum sea level, and the z axis is defined as the zenith direction in the frame. The wave superposition model which defines the geometry of the three-dimensional sea surface can be described as follows [13]:

(6) $$z=H\left(x,y,t\right)=\overset{m}{\underset{i=0}{\sum }}\overset{n}{\underset{j=0}{\sum }}a_{ij}\cdot \cos\left(k_{i}x\cos\theta_{j}+k_{i}y\sin\theta_{j}-\omega_{i}t-\varepsilon_{ij}\right)$$

where z=H(x,y,t) is the wave height of point (x,y) at time t; m is the division number of the angular frequency range; n is the division number of the direction angle range. For a component wave, $a_{ij}$ is its amplitude, $\omega_{i}$ is its angular frequency, $k_{i}$ is the corresponding wave number, $\theta_{j}$ is its direction angle, and $\varepsilon_{ij}$ is the random initial phase ($0\leq \varepsilon_{ij}\leq 2\pi$).

When we get the parameters of the component waves, we can obtain the shape of the wave under different wind conditions. For a specific wind condition, the spectral peak frequency $\omega_{m}$ is firstly obtained according to Equation (2), then we can select an angular frequency range $\left[\omega_{l},\omega_{h}\right],\omega_{l}<\omega_{m}<\omega_{h}$ correspondingly, and equally divide the frequency range to get the angular frequency of each component wave:

(7) $$\omega_{i}=\omega_{l}+i\cdot \Delta \omega ,i=0,1,2,\ldots ,m$$

where $\omega_{i}$ denotes the angular frequency of a component wave and Δω is the division increment of angular frequencies which satisfies $m\cdot \Delta \omega =\omega_{h}-\omega_{l}$. Meanwhile, wave number $k_{i}$ and angular frequency $\omega_{i}$ satisfy the following relation [14]:

(8) $$k_{i}=\frac{\omega_{i}^{2}}{g}$$

Although the theoretical variation range of the direction angle is [−π,π], the actual wave energy is mostly distributed within $\pm \frac{\pi }{2}$ on both sides of the wind direction. Representing the wind direction by U, we can equally divide the range $\left[U-\frac{\pi }{2},U+\frac{\pi }{2}\right]$ to get the direction angle of each component wave:

(9) $$\theta_{j}=U-\frac{\pi }{2}+j\cdot \Delta \theta ,\;j=0,1,2,\ldots ,n$$

where $\theta_{j}$ denotes the direction angle of a component wave and Δθ is the division increment of direction angles which satisfies n·Δθ=π.

According to Equation (5), $a_{ij}$ can be obtained as [13]:

(10) $$a_{ij}=\sqrt{2S\left(\omega_{i},\theta_{j}\right)\cdot \Delta \omega \cdot \Delta \theta }$$

Finally, the heights of every point on the wave surface can be calculated by combining Equations (6–10), so we can obtain the shape of the wave at any time under different wind conditions.

After investigation and comparison with typical actual wind conditions given by Beaufort wind force scale, we determined the ranges of angular frequency, the division increments and division numbers correspondingly, as shown in Table 1.

#### 2.1.2. Normal Vector of the Sea Surface

As we know, the normal vector of the point $M_{0}\left(x_{0},y_{0},z_{0}\right)$ on a three-dimensional surface S:z=f(x,y) is:

(11) $$\vec{N}\left(x_{0},y_{0},z_{0}\right)=\left(-f_{x}\left(x_{0},y_{0}\right),-f_{y}\left(x_{0},y_{0}\right),1\right)$$

where $\vec{N}\left(x_{0},y_{0},z_{0}\right)$ denotes the normal vector; $f_{x}\cdot \left(x_{0},y_{0}\right)={\frac{\partial z}{\partial x}|}_{\begin{array}{c} x=x_{0}\\ y=y_{0} \end{array}},\;f_{y}\cdot \left(x_{0},y_{0}\right)={\frac{\partial z}{\partial y}|}_{\begin{array}{c} x=x_{0}\\ y=y_{0} \end{array}}.$

For the sea surface S:z=H(x,y,t) defined by Equation (6), the following equation can be derived based on Equation (11):

(12) $$H_{x}\left(x_{0},y_{0},t\right)={\frac{\partial z}{\partial x}|}_{\begin{array}{c} x=x_{0}\\ y=y_{0} \end{array}}=-\overset{m}{\underset{i=0}{\sum }}\overset{n}{\underset{j=0}{\sum }}a_{ij}\cdot \sin\left(k_{i}x_{0}\cos\theta_{j}+k_{i}y_{0}\sin\theta_{j}-\omega_{i}t-\varepsilon_{ij}\right)\cdot k_{i}\cos\theta_{j}$$

(13) $$H_{y}\left(x_{0},y_{0},t\right)={\frac{\partial z}{\partial x}|}_{\begin{array}{c} x=x_{0}\\ y=y_{0} \end{array}}=-\overset{m}{\underset{i=0}{\sum }}\overset{n}{\underset{j=0}{\sum }}a_{ij}\cdot \sin\left(k_{i}x_{0}\cos\theta_{j}+k_{i}y_{0}\sin\theta_{j}-\omega_{i}t-\varepsilon_{ij}\right)\cdot k_{i}\sin\theta_{j}$$

Equations (12) and (13) shows that it is unavoidable to superimpose waves two times to calculate the two partial derivatives in order to obtain $\vec{N}\left(x_{0},y_{0},z_{0}\right)$. Moreover, the calculation of the two partial derivatives requires 2×m×n additional multiplication of $k_{i}$ and $\sin\theta_{j}$ (or $\cos\theta_{j}$). Generally, the extra computation consumption is very considerable especially when m and n are relatively large. To avoid this problem, we obtained the normal vector of a point on the wave surface by calculating the sum of the normal of the adjacent meshes rather than by using the analytical computation given by Equations (11)–(13).

**Figure 2:**
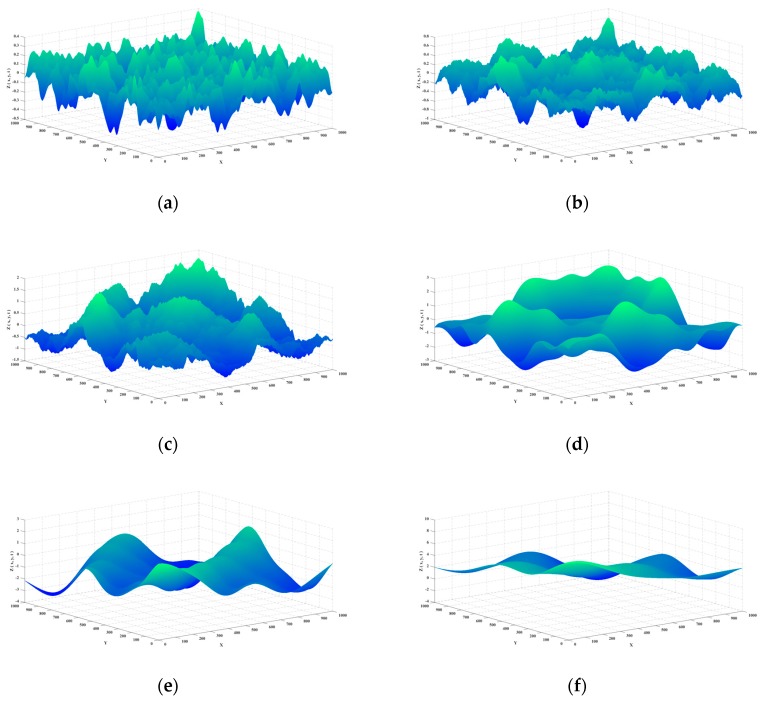
1000 × 1000 mesh of the waves at typical wind speeds. (**a**) the wind speed is 5 m/s; (**b**) the wind speed is 7 m/s; (**c**) the wind speed is 9 m/s; (**d**) the wind speed is 13 m/s; (**e**) the wind speed is 16 m/s; (**f**) the wind speed is 20 m/s.

The x−y plane region of a certain size is divided into M×N uniform regular mesh by the step size q. For each mesh node $\left(x_{i},y_{j}\right),i=1,2,\ldots ,M;j=1,2,\ldots ,N$, the wave height $z_{i,j}=H\left(x_{i},y_{j},t\right)$ can be calculated according to Equation (6). In this way, an approximate sea surface with M×N mesh nodes is determined. The larger the M and N are, the denser the mesh is, and the smoother the surface is. As Figure 2 shows, 1000 × 1000 mesh of the waves at typical wind speeds is drawn in the x−y−z three-dimensional coordinate system based on the parameters shown in Table 1.

**Figure 3:**
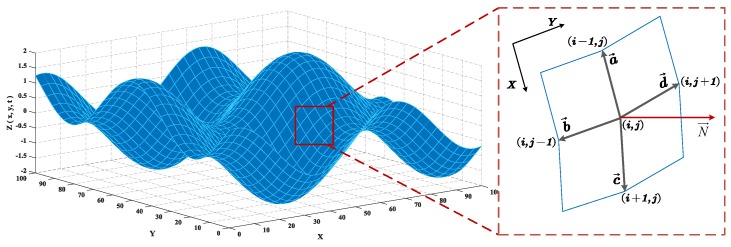
The mesh node and its four surrounding mesh elements.

Each mesh node $\left(x_{i},y_{j},z_{i,j}\right)$ on the wave surface is adjacent to four mesh nodes: $\left(x_{i-1},\;y_{j},z_{i-1,j}\right),\left(x_{i},\;y_{j+1},z_{i,j+1}\right),\left(x_{i+1},\;y_{j},z_{i+1,j}\right),\left(x_{i},\;y_{j-1},z_{i,j-1}\right)$, and $\left(x_{i},y_{j},z_{i,j}\right)$ is the intersection of the four surrounding mesh elements, each of which has an independent normal vector, as shown in Figure 3.

In Figure 3, $\vec{a}$, $\vec{b}$, $\vec{c}$, and $\vec{d}$ are the common edge vectors of the four mesh elements respectively:

(14) $$\left\{ \begin{array}{l} \vec{a}=\left(x_{i-1}-x_{i},\;y_{j}-y_{j},z_{i-1,j}-z_{i,j}\right)=\left(-q,0,z_{i-1,j}-z_{i,j}\right)\\ \vec{b}=\left(x_{i}-x_{i},\;y_{j-1}-y_{j},z_{i,j-1}-z_{i,j}\right)=\left(0,-q,z_{i,j-1}-z_{i,j}\right)\\ \vec{c}=\left(x_{i+1}-x_{i},\;y_{j}-y_{j},z_{i+1,j}-z_{i,j}\right)=\left(q,0,z_{i+1,j}-z_{i,j}\right)\\ \vec{d}=\left(x_{i}-x_{i},\;y_{j+1}-y_{j},z_{i,j+1}-z_{i,j}\right)=\left(0,q,z_{i,j+1}-z_{i,j}\right) \end{array}\right.$$

The normal vectors of the four surrounding mesh elements are cross-products of $\vec{a}$ and $\vec{b}$, $\vec{b}$ and $\vec{c}$, $\vec{c}$ and $\vec{d}$, and $\vec{d}$ and $\vec{a}$ respectively. The sum of the four normal vectors is taken as the normal vector of the mesh node $\left(x_{i},y_{j},z_{i,j}\right)$:

(15) $$\vec{N}\left(x_{i},y_{j},z_{i,j}\right)=\left(\vec{a}\times \vec{b}+\vec{b}\times \vec{c}+\vec{c}\times \vec{d}+\vec{d}\times \vec{a}\right)$$

In the above calculation process, only one wave superposition is needed for each mesh node to get the wave heights, and the normal vector can be calculated by using the height values of adjacent mesh nodes, which avoids a lot of additional computation. The denser the mesh is, the closer the normal vectors obtained by this method is to those obtained by the analytical computation.

### 2.2. Modeling Transmittance at Different Incidence Angle

#### 2.2.1. Incidence Angle Modeling

For a typical LiDAR detection scene, an aircraft is generally used as the air platform to carry the blue-green laser emitter and detector. The transmission of the blue-green laser on the sea surface includes two parts: the down channel where laser pulse signals are transmitted from the air to the underwater, and the up channel where the reflected pulse echoes are transmitted in the opposite direction.

**Figure 4:**
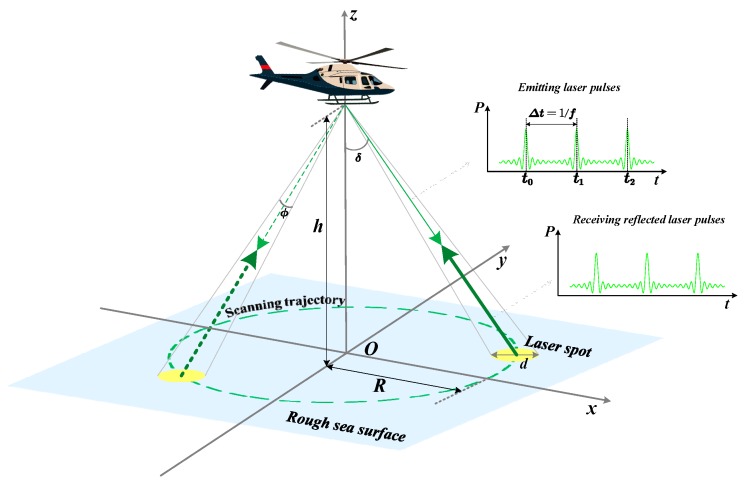
A typical LiDAR detection scene.

During the continuous flight of the aircraft, the emitter sent the laser pulses along the down channel in the form of circular scanning. Due to the existence of inherent divergence angle of the laser, a pulse was projected on the sea surface to form a spot in the form of laser beam. Therefore, it was necessary for us to focus on the spot to analyze and calculate the transmittance. After the laser beam was transmitted through the sea surface, it hits the underwater target surface by which the reflected pulse echo was formed. Next, the reflected pulse echo was received by the detector along the up channel through the surface. Figure 4 shows the schematic diagram of a typical scene of LiDAR scanning detection.

As shown in Figure 4, the motion and scanning of airborne LiDAR over the sea surface were quantitatively described in a right-hand three-dimensional coordinate frame which was consistent with the previous one used to describe the three-dimensional sea surface. The x−y plane denotes the horizontal datum sea level and the vertical z axis faced upward; O indicates the initial horizontal position of the aircraft and h represents the fixed flight altitude of the aircraft. In Figure 4, the scanning trajectory was circular, while the actual scanning trajectory on the sea surface was helix due to the move of the aircraft; δ denotes the laser scanning angle, which is the angle between the laser emission direction and the z axis and ϕ is the laser beam divergence angle. The laser pulses were emitted every Δt at the frequency of $f_{emit}$($\Delta t=1/f_{emit}$), and each laser pulse was projected on the sea surface to form a spot. Because ϕ is generally very small, the pulse spot could be approximately regarded as a solid circle, and its diameter d could be approximately obtained by the following formula:

(16) $$d=2\cdot \frac{h}{\cos\delta }\cdot \tan\left(\frac{\phi }{2}\right)\approx \frac{h\phi }{\cos\delta }$$

The radius of the scanning trajectory R can be obtained as:

(17) R=h·tanδ

**Figure 5:**
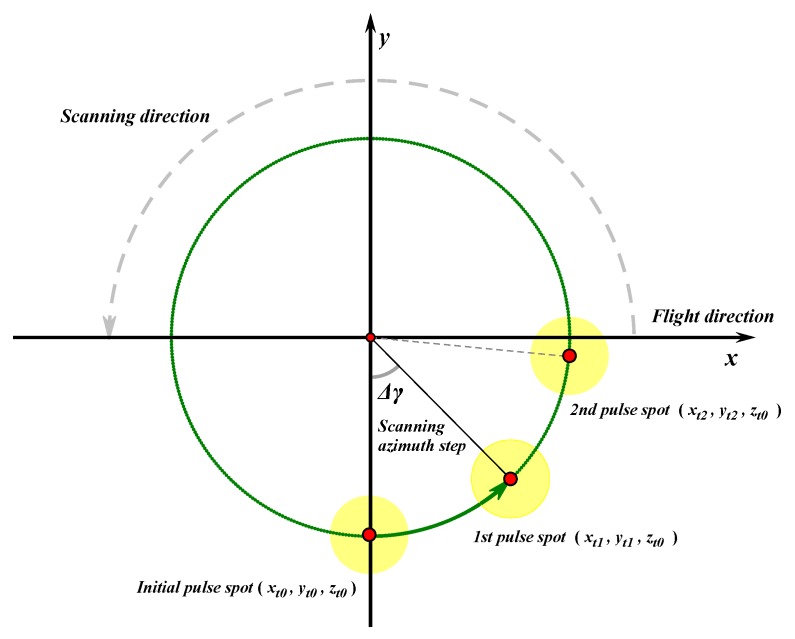
The top view of laser scanning detection.

Figure 5 shows the laser scanning process from the top view. For the convenience of analysis, the plane was set to fly in the positive direction of the x axis with a flight speed of v; The LiDAR was scanning in the counterclockwise direction with a scanning frequency of $f_{scan}$ in revolutions per second, which indicates the number of scanning trajectory circles per second.

In Figure 5, the coordinate of a pulse spot center is used to represent the pulse spot position; Δγ denotes the scanning azimuth step between the next pulse spot and the previous pulse spot can be obtained as:

(18) $$\Delta \gamma =2\pi \cdot f_{scan}\cdot \Delta t=\frac{2\pi f_{scan}}{f_{emit}}$$

We set the aircraft coordinate at the initial time $t_{0}$ as $\left(X_{t0}^{c},Y_{t0}^{c},h\right)$, and let the pulse spot coordinate be $\left(x_{t0},y_{t0},\;z_{t0}\right)$ correspondingly. There was an obvious relationship as follows.

(19) $$\{\begin{array}{l} x_{t0}=X_{t0}^{c}\\ y_{to}=Y_{t0}^{c}-R\\ z_{to}=H\left(x_{t0},y_{t0},t_{0}\right) \end{array}$$

We let the successive aircraft coordinate at the time $t_{i}\left(t_{i}=t_{0}+i\cdot \Delta t\right)$ be $\left(X_{ti}^{c},Y_{ti}^{c},h\right)$, and let the pulse spot coordinate be $\left(x_{ti},y_{ti},z_{ti}\right)$ correspondingly. Because the aircraft was set to fly in the positive direction of the x axis, the following relationship can be easily obtained as:

(20) $$\{\begin{array}{l} X_{ti}^{c}=X_{t0}^{c}+v\cdot i\cdot \Delta t\\ Y_{ti}^{c}=Y_{t0}^{c} \end{array}$$

According to the angle relationship, considering the displacement caused by the aircraft flight, $\left(x_{ti},y_{ti},z_{ti}\right)$ can be derived as follows:

(21) $$\{\begin{array}{l} x_{ti}=x_{t0}\cos\left(i\cdot \Delta \gamma \right)-y_{to}\sin\left(i\cdot \Delta \gamma \right)+v\cdot i\cdot \Delta t\\ y_{ti}=x_{t0}\sin\left(i\cdot \Delta \gamma \right)+y_{to}\cos\left(i\cdot \Delta \gamma \right)\\ z_{ti}=H\left(x_{ti},y_{ti},t_{i}\right) \end{array}$$

Substituting Equations (18) and (19) into Equation (21), the following expression can be obtained as:

(22) $$\left\{ \begin{array}{l} x_{ti}=X_{t0}^{c}\cos\left(\frac{2\pi if_{scan}}{f_{emit}}\right)-\left(Y_{t0}^{c}-R\right)\sin\left(\frac{2\pi if_{scan}}{f_{emit}}\right)+\frac{vi}{f_{emit}}\\ y_{ti}=X_{t0}^{c}\sin\left(\frac{2\pi if_{scan}}{f_{emit}}\right)+\left(Y_{t0}^{c}-R\right)\cos\left(\frac{2\pi if_{scan}}{f_{emit}}\right)\\ z_{ti}=H\left(x_{ti},y_{ti},t_{i}\right) \end{array}\right.$$

**Figure 6:**
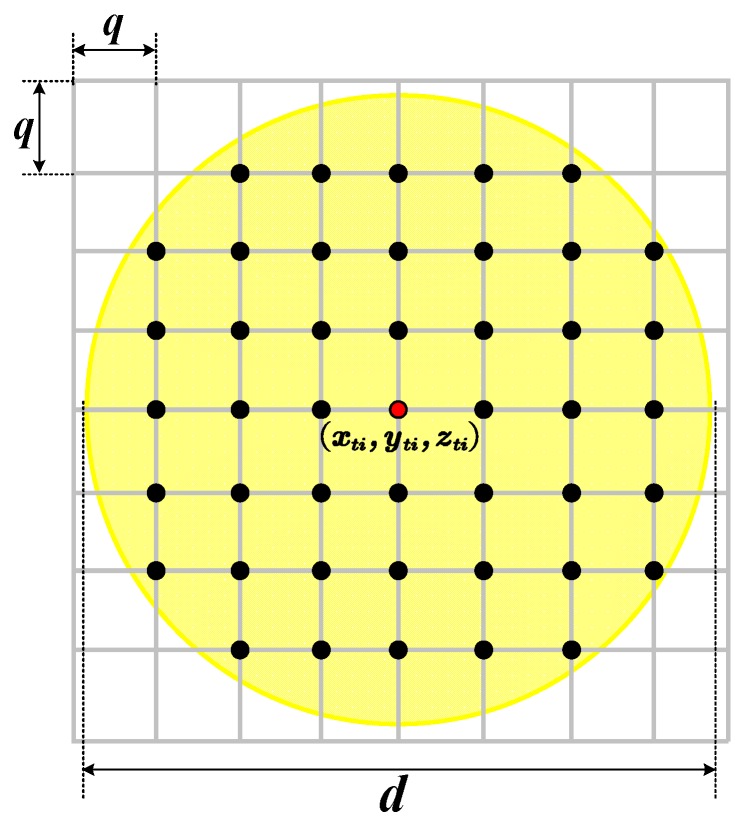
Mesh nodes inside the pulse spot.

After obtaining the coordinate of the pulse spot, we focused on the local wave mesh at the pulse spot position. As Figure 6 shows, multiple mesh nodes are covered by the pulse spot at time $t_{i}$. Next, we began to analyze the transmission characteristics of the mesh nodes inside the pulse spot. 

The mesh nodes inside the pulse spot at time $t_{i}$ form a point set $I_{ti}$ and shown as:

(23) $$I_{ti}:\left\{\left(x_{p},y_{q},z_{p,q}\right)|{\left[\left(x_{p}-x_{ti}\right)q\right]}^{2}+{\left[\left(y_{q}-y_{ti}\right)q\right]}^{2}\leq {\left(\frac{d}{2}\right)}^{2};z_{p,q}=H\left(x_{p},y_{q},t_{i}\right)\right\}$$

where $\left(x_{p},y_{q},z_{p,q}\right)$ indicates the coordinates of these internal nodes; d is the spot diameter given by Equation (16); and q is the step size of the wave mesh mentioned in Section 2.1.2.

**Figure 7:**
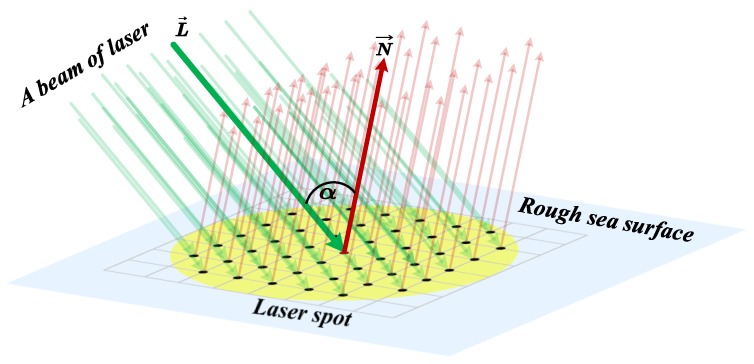
The incident angles of the laser light in a laser beam.

Figure 7 shows the incident angles of the laser light in a laser beam. Naturally, for each laser light line in the laser beam, the falling point of them were considered different. However, only those light lines falling on the mesh nodes were considered. $\vec{L}$ denotes the vector of the laser light line falling on a node; $\vec{N}$ is the normal vector of the node; and α is the incident angle of the laser light line.

For every mesh node in point set $I_{ti}$, the vector of the light line falling on the node can be obtained as:

(24) $$\vec{L}\left(x_{p},y_{q},z_{p,q}\right)=\left(x_{p}-X_{ti}^{c},y_{p}-Y_{ti}^{c},z_{p,q}-h\right)$$

Combining with the normal vector of the mesh node given by Equation (15), the incident angle of the light line falling on $\left(x_{p},y_{q},z_{p,q}\right)$ can be obtained as:

(25) $$\alpha \left(x_{p},y_{p},z_{p,q}\right)=\frac{\left|\vec{L}\left(x_{p},y_{q},z_{p,q}\right)\cdot \vec{N}\left(x_{p},y_{p},z_{p,q}\right)\right|}{\left|\vec{L}\left(x_{p},y_{q},z_{p,q}\right)\right|\cdot \left|\vec{N}\left(x_{p},y_{p},z_{p,q}\right)\right|}$$

#### 2.2.2. Transmittance Modeling

Because the wavelength of the blue-green laser (470–580 nm) was far less than the millimeter wave band where the wave characteristic wavelength was located [15], the geometrical optics theory based on the Fresnel formula was applicable to the analysis of the transmission characteristics on the interface. 

The refraction occurred when the laser was transmitted through the sea surface twice along the down channel and the up channel respectively. The wave motion may change the light path in this short time between the two transmissions. For the down channel, the transmission medium of the refracted light was sea water. We let the length of the underwater transmission path between the incident point and the target be L. The physical definition of the optical path:

(26) $$L_{r}=n_{SA}\cdot L$$

where $L_{r}$ denotes the optical path of the refracted light in sea water and $n_{SA}$ is the refractive index of the sea water relative to the air ($n_{SA}=1.33$).

At present, the depth of the underwater target is within 100m. We set L=100 m and the light speed in vacuum $c=3\times 10^{8}\;m/s$. The time difference Δt between the two transmissions through the sea surface can be calculated as:

(27) $$\Delta t=\frac{2\cdot L_{r}}{c}\approx 0.89\;\mu s$$

The phase difference Δφ of the wave between the two transmissions can be obtained as:

(28) $$\Delta \varphi =\frac{\Delta t}{T_{s}}\cdot 2\pi$$

where $T_{s}$ is the average period of the wave motion, generally at 1–9 s according to the observation and statistics [16,17,18,19]. Substituting the range of $T_{s}$ into Equation (28), the corresponding range of Δφ can be obtained as:

(29) $$1.98\times 10^{-7}\pi <\Delta \varphi <1.78\times 10^{-6}\pi$$

**Figure 8:**
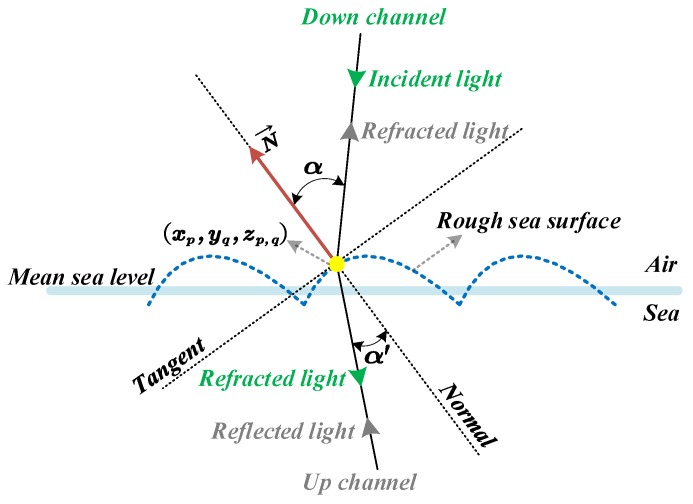
Schematic diagram of the light path of the down channel and the up channel.

It can be seen that Δφ was extremely small, so the motion of the wave in this period can be ignored. It is considered that the wave was practically stationary in this time between the two transmissions. As a result, the light paths of the down channel and the up channel were reversed, and the laser light lines along the two channels passed through the same incident point on the rough sea surface, which is shown in Figure 8.

In Figure 8, $\left(x_{p},y_{q},z_{p,q}\right)$ is a mesh node inside the pulse spot on the interface, as the same incident point of the laser light lines along the down channel and up channel respectively; For the down channel, α represents the angle of incidence, and $\alpha^{\prime }$ represents the angle of refraction; On the contrary, α represents the angle of refraction and $\alpha^{\prime }$ represents the angle of incidence for the up channel. We can analysis the transmittance based on α which can be obtained by Equation (25).

The Snell Law gives a relationship between α and $\alpha^{\prime }$ as follows:

(30) $$\frac{\sin\left(\alpha \right)}{\sin\left(\alpha^{\prime }\right)}=n$$

When the laser is transmitted from the first medium to the second medium, it could be decomposed into two mutually perpendicular components, the ***S*** wave and ***P*** wave. The ***S*** wave was the light component perpendicular to the incident plane and the ***P*** wave was the one parallel to the incident plane. The amplitude transmission coefficients of the ***S*** wave and ***P*** wave were given by the Fresnel formula as follows:

(31) $$\{\begin{array}{l} t_{s}=\frac{2\sin\theta_{y}\cos\theta_{x}}{\sin\left(\theta_{x}+\theta_{y}\right)}\\ t_{p}=\frac{2\sin\theta_{y}\cos\theta_{x}}{\sin\left(\theta_{x}+\theta_{y}\right)\cdot \cos\left(\theta_{x}-\theta_{y}\right)} \end{array}$$

where $t_{s}$ and $t_{p}$ are the amplitude transmission coefficients of the ***S*** wave and ***P*** wave respectively; $\theta_{x}$ is the incident angle in the first medium, and $\theta_{y}$ is the refraction angle in the second medium.

Considering the polarization of the laser light, and according to the Malus Law, the transmittance can be calculated as follows:

(32) $$\rho =\frac{n_{2}}{n_{1}}\cdot \frac{\cos\theta_{y}}{\cos\theta_{x}}\cdot \left(t_{s}^{2}\cdot \sin^{2}\beta +t_{p}^{2}\cdot \cos^{2}\beta \right)$$

(33) $$\beta^{\prime }=\arctan\left(\frac{t_{s}}{t_{p}}\cdot \tan\beta \right)=\arctan\left[\cos\left(\theta_{x}-\theta_{y}\right)\cdot \tan\beta \right]$$

where ρ is the transmittance of the refracted light energy to the incident light energy; $n_{1}$ and $n_{2}$ are the refractive index of the first medium and the second medium respectively; and β represents the polarization azimuth of the incident light relative to the incident plane. $\beta^{\prime }$ represents the polarization azimuth of the refracted light relative to the incident plane.

According to Figure 8, for the down channel, the following equation can be obviously obtained:

(34) $$\{\begin{array}{l} \theta_{y}=\alpha \\ \theta_{x}=\alpha^{\prime }\\ \frac{n_{2}}{n_{1}}=n \end{array}$$

In the case that the reflection characteristics of the target surface was not available, it was assumed that the specular reflection occurred on the surface of the underwater target. As a result, the polarization characteristics of reflected light were not changed. Therefore, for the up channel, the corresponding equation is:

(35) $$\{\begin{array}{l} \theta_{y}=\alpha^{\prime }\\ \theta_{x}=\alpha \\ \frac{n_{2}}{n_{1}}=\frac{1}{n}\\ \beta =\beta^{\prime } \end{array}$$

Considering Equations (30)–(35) comprehensively, the transmittance of the laser light line passing through the mesh node $\left(x_{p},y_{q},z_{p,q}\right)$ along the down channel and the up channel could be calculated respectively. Furthermore, the total transmittance at $\left(x_{p},y_{q},z_{p,q}\right)$ can be obtained by multiplying them as follows.

(36) $$\rho \left(x_{p},y_{q},z_{p,q}\right)=\rho_{d}\left(x_{p},y_{q},z_{p,q}\right)\cdot \rho_{u}\left(x_{p},y_{q},z_{p,q}\right)$$

where $\rho \left(x_{p},y_{q},z_{p,q}\right)$ denotes the total transmittance; $\rho_{d}\left(x_{p},y_{q},z_{p,q}\right)$ denotes the transmittance of the down light line, and $\rho_{u}\left(x_{p},y_{q},z_{p,q}\right)$ denotes the transmittance of the up laser light line.

For the whole pulse spot on the rough sea surface, the average value of the transmittance at all mesh nodes inside the spot was taken as the transmittance of the spot, as follows:

(37) $$\rho_{spot}=\frac{1}{N}\overset{N}{\underset{i=1}{\sum }}\rho \left(x_{p},y_{q},z_{p,q}\right)$$

where $\rho_{spot}$ denotes the transmittance of the pulse spot and N denotes the number of the mesh nodes inside the pulse spot.

## 3. Simulations and Results

Based on the models and simulation methods presented in Section 2, several simulations for the pulse spot transmittance and refraction angles under different wind conditions were performed. Section 3.1 described simulation conditions and parameters. Section 3.2 compared the simulation speeds of the proposed method with parallel computing with those of the traditional analytical method with sequential computing. Section 3.2 provided the statistics of the pulse spot refraction angles and transmittance.

### 3.1. Simulation Conditions

We set the coordinate of the aircraft at initial time as (0,0,h); The step size of the mesh is set as q=0.01 m; In the first 100 s, we took a pulse spot every 0.1 s, and computed the transmittance of the 1000 pulse spots in different typical wind speeds and wind directions. The simulation environment is shown in Table 2. We took the working condition parameters of mainstream airborne LiDAR systems as the simulation parameters, as shown in Table 3.

### 3.2. Simulation Time 

In the simulation, we first used the traditional differential analytic method to calculate the normal vector of the sea surface, and simulated the transmission of 1000 pulse spots with sequential computing. To illustrate the rapidity of the simulation method, we then used the mesh division method used in this paper to calculate the normal vector, and simulated the transmission of the pulse spots with parallel computing. The simulation time comparison between the two methods is shown in the Table 4. In simulation, typical wind speeds were taken from Beaufort wind force scale to simulate various wind conditions (include strong wind), which may be encountered in practical application.

From Table 4, it can be seen that the proposed meshing method with parallel computing used in this paper can reduce the simulation time consumption by about 70% compared with the traditional analytical method with sequential computing.

### 3.3. Statistics of Refraction Angles

The refraction angles on the mesh points in a pulse spot were averaged as the refraction angle of the pulse spot. The maximum, minimum, and average of the refraction angles of 1000 pulse spots were obtained, as shown in Table 5.

**Figure 9:**
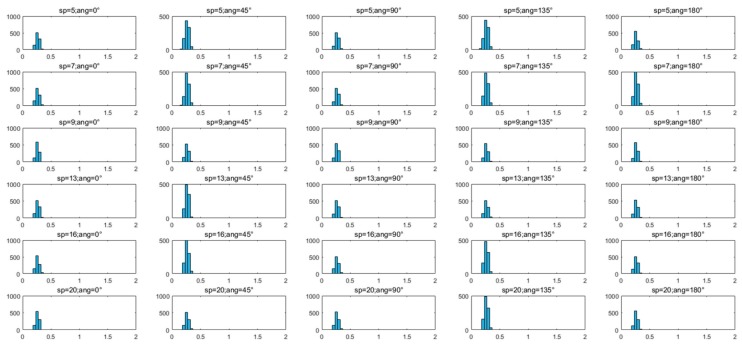
Histogram of refraction angle frequency distribution of 1000 pulse spots.

The histogram of refraction angle frequency distribution of the 1000 pulse spots is shown in Figure 9.

From the statistical results of the refraction angles of pulse spots shown in Table 5, we can see that the refraction angles under each wind condition were concentrated between 0.14 rad and 0.38 rad; Furthermore, Figure 9 shows that the distributions of refraction angles under each wind condition were consistent with the Gaussian distribution. For each wind condition, more than 90% of the pulse spot refraction angles were distributed in three bins of [0.1848 rad, 0.3234 rad], and the maximum frequency was in the bin of [0.2310 rad, 0.2772 rad].

From the results, we can see the dynamic range of refraction angle was relatively small. However, the planimetric effects and depth coordinate errors caused by the refraction angle were considerable to detect the underwater targets at tens or even hundreds of meters [20]. Therefore, it is necessary to quickly calculate the refraction angle under different wind conditions to correct the detection results.

### 3.4. Statistics of the Transmittance

In the practical application of airborne LiDAR, the minimum value of transmittance should be considered in the detection, which decides the maximum detection depth. The maximum value of transmittance should be considered in the anti-detection, which decides the minimum safe depth. The maximum, minimum and average of the transmittance of 1000 pulse spots were obtained, as shown in Table 6.

**Figure 10:**
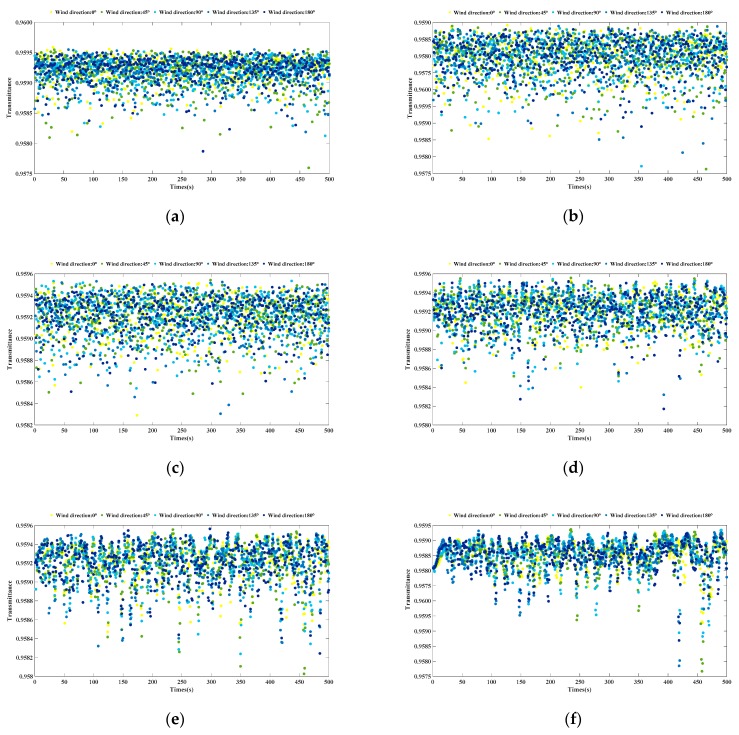
Scatter distribution of the transmittance of 1000 pulse spots under different wind conditions. (**a**) The wind speed is 5 m/s; (**b**) the wind speed is 7 m/s; (**c**) the wind speed is 9 m/s; (**d**) the wind speed is 13 m/s; (**e**) the wind speed is 16 m/s; (**f**) the wind speed is 20 m/s.

The scatter diagram of the transmittance of the 1000 pulse spots are shown in Figure 10.

From the statistical results of the pulse spot transmittance shown in Table 6, we can see that the pulse spot transmittance at different positions under each wind condition had an average value of about 95.9%; Furthermore, as Figure 10 shows, it is apparent that the distribution of the pulse spot transmittance under different wind conditions was similar, and the transmittance values were concentrated between 95.7% and 96%. 

## 4. Discussion

In this paper, we analyzed and computed laser transmittance through the rough wind-driven sea surface and we obtained some statistical properties of the transmittance of pulse spots under different wind conditions. As the statistical results shows, the refraction angles of pulse spots were concentrated between a small range, and the transmittance of pulse spots was generally high and stable under different wind conditions. The results may be explained as follows.

**Figure 11:**
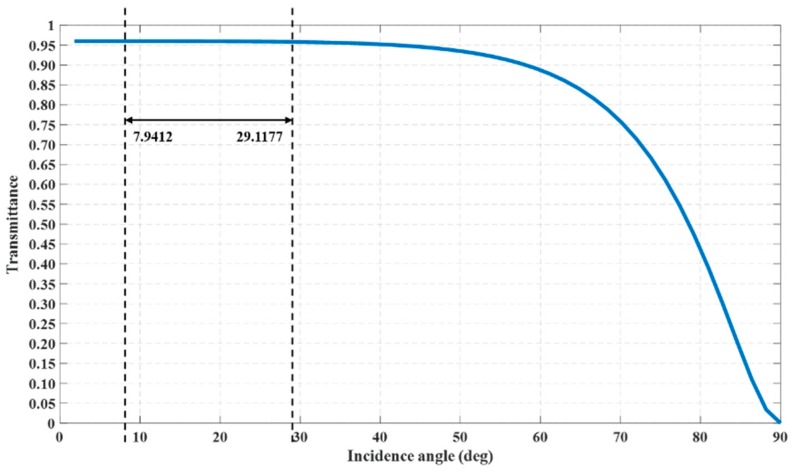
The relationship between transmittance and the incident angle of the down channel.

Firstly, we can obtain the relationship between the transmittance and the incident angle of the down channel based on the computational process given by Equations (30)–(36), as shown in Figure 11.

It can be seen that the transmittance was above 95% and was almost unchanged when the incident angle changed in the range of about 0°~40°. Meanwhile, when the incident angle increased to 57°, the transmittance slowly decreases to 90%; When the incident angle increased in the range of 60°~90°, the transmittance decreased rapidly to 0. According to our calculation results, the incident angles of the pulse spots were concentrated in the range of 7.9412° to 29.1177°, which meant that the transmittance and the refraction angles are correspondingly distributed in a small range. 

Furthermore, under the set simulation conditions, according to the actual working conditions of the LiDAR detection, the size of the pulse spot on the sea surface was about 26 cm in diameter, which was much smaller than the scale of the wave. There was little difference in the incident angles of the light lines falling on every mesh node inside the pulse spot. Therefore, the transmittance of a pulse spot was quite close to that of the mesh nodes inside it, which depended on the incident angle. In addition, because of the relatively small rotation scanning angle, the incident angle was almost determined by the inclination of the fluctuating sea surface.

First of all, under low wind speeds, the sea surface was gentle where there was basically no fluctuation, so the inclination of the sea surface was relatively small. Furthermore, with the increase of the wind speed above the sea surface, the larger wave energy described based on the PM spectrum was concentrated in a smaller frequency band. Consequently, the fluctuation of the rough sea surface became much larger, which meant that the wave height in the vertical direction and the wave scale in the horizontal direction became larger at the same time, so the inclination of the sea surface remained relatively small. Moreover, because the wave fluctuates periodically throughout the horizontal plane, the overall range of the sea surface inclination hardly changeed with the wind direction which only affected the distribution of the sea surface inclination at different positions. Therefore, in the description of the PM wave spectrum, the wind speed and directions had little effect on the inclination of the sea surface.

As a result, the incident angles of the down channel in the scanning process were small. Therefore, the simulation results were reasonable and consistent with the conclusion of the research of Dong et al. [21]. The effects of low wind speeds from 2 to 5.25 m/s on the refraction angle were analyzed in Reference [8]. From Figure 10 in Reference [8], it can be seen that no correlation was observed between the means of the laser beam center (refraction angles) and wind speeds. The experimental results are coincident with those in our paper.

However, it should be noted that the outcome of this study cannot be taken as evidence that complicated wind conditions will never affect the transmittance and refraction angle on the sea surface, because we did not consider other relevant factors on the dynamic complex sea surface, such as foams, tides, other random fluctuations, and special wave patterns beyond the description range of the PM spectrum. Specifically speaking, foams covered on the sea surface can significantly enhance surface scattering with the increase of wind speed, which has a great influence on the transmittance [9]. The tides and random fluctuations can increase the geometry uncertainty of the sea surface [10,20], which may greatly affect the transmission path of the laser to influence the transmittance and refraction angles. Therefore, there is abundant room for further progress in establishing a more unified and comprehensive model for more complete and complex environmental conditions by introducing other environmental factors. Previous relevant research may serve as a necessary basis for further study [9,10,22,23].

This finding provides some statistical laws of refraction angles and transmittance through the fluctuating sea surface under complex wind conditions, which may serve as a basis for future fast computation of airborne LiDAR transmission in complex environments. Moreover, this preliminary finding has a certain reference significance for the correlative development, verification, and practical application of the LiDAR. From the perspective of detecting, benefiting from the high concentration of the energy inside the pulse spot, the echo strength, and the detection depth of the LiDAR are almost unaffected by the environment on the sea surface. From the perspective of anti-detecting, the windy weather hardly reduces the risk detected by the LiDAR on account of the invariably guaranteed detection effect. With this fast analysis method, the transmission characteristic in the current complex environment can be quickly found out in order to undertake corresponding correction and anti-detection.

## 5. Conclusions

This paper proposes a fast analysis method for blue-green laser transmission through a wind-driven dynamic sea surface. We obtained the transmission characteristic under different wind speeds and directions quickly with the meshing method and parallel calculation method. The simulation results show that our method can reduce the time consumption by 70% compared with the traditional method. The statistical results suggest that the refraction angles and transmittance of pulse spots on the fluctuating rough sea surface is generally stable under different wind conditions, which may provide a basis for the future fast computation of airborne LiDAR transmission in complex environments. Meanwhile, this study provided a fresh and useful mean for the fast analysis of the transmission process of the LiDAR detection, which may have a certain reference significance for the development, verification, and practical application of the LiDAR. 

However, one possible limitation involves the actual transmission process, which is extremely complicated and, is not affected only by the sea wind, but rather a variety of coupled environmental factors. One direction of the future work is to take other environmental factors, such as foams, tides, and other random fluctuations, into account in order to analyze more comprehensive transmission mechanisms on the complex sea surface. Furthermore, our study only focuses on the simulation of parameters of mainstream airborne LiDAR systems, and gives some reference conclusions. In future research, it might be possible to conduct more investigations for the relationship of more LiDAR system parameters and their random and systematic effects on the detection results.

## Figures and Tables

**Table 1:** Simulation parameters of the three-dimensional waves under typical wind speeds.

Wind Scale ^1^	Wave Grade ^1^	u (m/s)	$\omega_{m}$ (rad/s)	$\left[\omega_{l},\omega_{h}\right]$ (rad/s)	Δω (rad/s)	m	Δθ (rad)	n
3	Micro waves	5	1.71	1.2~5.0	0.2	20	π/5	7
4	Small waves	7	1.22	0.6~4.0	0.2	18	π/10	11
5	Moderate waves	9	0.95	0.5~2.5	0.1	21	π/10	11
6	Large waves	13	0.66	0.4~2.0	0.1	17	π/20	11
7	Strong waves	16	0.54	0.3~1.6	0.05	27	π/20	21
8–9	High waves	20	0.43	0.2~1.2	0.05	21	π/20	21

^1^ Given by the Beaufort wind force scale.

**Table 2:** Simulation experiment environment.

Central Processing Unit (CPU)	Random Access Memory (RAM)	Graphics Processing Unit (GPU)	Integrated Development Environment (IDE)	Parallel Pool
Intel(R) Core(TM) i7-7700 (3.60GHz)	32 GB	NVIDIA GeForce GTX 1060 (6 GB)	Matlab R2016b	4 cores

**Table 3:** Simulation parameters.

h (m)	δ (°)	ϕ (mrad)	v (km/h)	$f_{emit}\;(\mathrm{Hz})$	$f_{scan}\;(\mathrm{Hz})$
500	20	0.5	300	55k	10

**Table 4:** Simulation time of the transmission of 1000 pulse spots.

Wind Speed (m/s)	Wind Direction (deg)	Time of Differential Analytic Method (s)	Time of the Proposed Method (s)	Time Reduction Proportion
5	0	25.72	8.46	67.11%
5	45	21.02	6.83	67.51%
5	90	13.86	3.89	71.94%
5	135	11.42	3.33	70.84%
5	180	16.06	4.39	72.67%
7	0	16.11	4.80	70.21%
7	45	21.25	6.27	70.49%
7	90	26.68	8.64	67.61%
7	135	21.58	6.95	67.80%
7	180	17.50	4.38	74.98%
9	0	11.79	3.49	70.40%
9	45	14.57	4.49	69.18%
9	90	15.96	4.89	69.35%
9	135	21.68	6.68	69.19%
9	180	34.24	9.23	73.04%
13	0	26.58	7.64	71.26%
13	45	14.94	4.06	72.82%
13	90	11.09	3.66	67.00%
13	135	16.95	4.95	70.79%
13	180	18.26	5.70	68.79%
16	0	24.88	6.91	72.23%
16	45	34.63	10.13	70.75%
16	90	31.12	8.39	73.04%
16	135	12.82	4.09	68.09%
16	180	11.96	3.81	68.16%
20	0	18.14	4.83	73.37%
20	45	20.34	5.38	73.55%
20	90	22.62	7.32	67.64%
20	135	40.72	10.96	73.09%
20	180	31.35	8.69	72.28%

**Table 5:** Statistical results of the refraction angles of 1000 pulse spots.

Wind Speed (m/s)	Wind Direction (deg)	Minimum (rad)	Maximum (rad)	Average (rad)
5	0	0.1702	0.3451	0.2618
5	45	0.1656	0.3539	0.2631
5	90	0.2034	0.3512	0.2650
5	135	0.1884	0.3446	0.2629
5	180	0.1918	0.3585	0.2618
7	0	0.1405	0.3602	0.2633
7	45	0.1626	0.3848	0.2654
7	90	0.1768	0.3760	0.2680
7	135	0.1540	0.3816	0.2652
7	180	0.1864	0.3800	0.2632
9	0	0.1506	0.3613	0.2646
9	45	0.1529	0.3738	0.2657
9	90	0.1564	0.3722	0.2667
9	135	0.1597	0.3648	0.2657
9	180	0.1583	0.3657	0.2647
13	0	0.1878	0.3514	0.2639
13	45	0.1704	0.3649	0.2645
13	90	0.1759	0.3639	0.2652
13	135	0.1634	0.3609	0.2645
13	180	0.1931	0.3572	0.2646
16	0	0.1432	0.3448	0.2649
16	45	0.1582	0.3716	0.2647
16	90	0.1627	0.3576	0.2648
16	135	0.1774	0.3648	0.2650
16	180	0.1719	0.3668	0.2650
20	0	0.1805	0.3594	0.2630
20	45	0.1611	0.3655	0.2637
20	90	0.1580	0.3543	0.2644
20	135	0.1547	0.3537	0.2642
20	180	0.1535	0.3540	0.2647

**Table 6:** Statistical results of the transmittance of 1000 pulse spots.

Wind Speed (m/s)	Wind Direction (deg)	Minimum	Maximum	Average
5	0	0.95847	0.95952	0.95921
5	45	0.95831	0.95951	0.95921
5	90	0.95850	0.95952	0.95920
5	135	0.95760	0.95955	0.95916
5	180	0.95785	0.95956	0.95917
7	0	0.95849	0.95954	0.95920
7	45	0.95852	0.95956	0.95920
7	90	0.95803	0.95955	0.95920
7	135	0.95767	0.95955	0.95920
7	180	0.95829	0.95948	0.95920
9	0	0.95812	0.95951	0.95917
9	45	0.95789	0.95953	0.95918
9	90	0.95854	0.95953	0.95919
9	135	0.95838	0.95954	0.95920
9	180	0.95824	0.95952	0.95919
13	0	0.95813	0.95954	0.95920
13	45	0.95840	0.95951	0.95920
13	90	0.95819	0.95955	0.95917
13	135	0.95805	0.95956	0.95917
13	180	0.95830	0.95953	0.95919
16	0	0.95832	0.95953	0.95919
16	45	0.95832	0.95956	0.95919
16	90	0.95774	0.95953	0.95919
16	135	0.95816	0.95950	0.95922
16	180	0.95787	0.95952	0.95919
20	0	0.95836	0.95952	0.95919
20	45	0.95851	0.95950	0.95920
20	90	0.95817	0.95953	0.95919
20	135	0.95824	0.95956	0.95919
20	180	0.95807	0.95951	0.95920
